# The Value of Ureteral Catheterisation and Cryoscopy of the Urine

**Published:** 1904-01-30

**Authors:** 


### THE VALUE OF URETERAL CATHETERISATION AND CRYOSCOPY OF THE URINE.

DR. W. E. LOWER[1] calls attention to the usefulness of these clinical methods in cases of renal disease where the question of operation arises. He points out that recent improvements in renal surgery have been in the direction of greater precision in diagnosis rather than in better technique. The most frequent cause of death after operation being incompetence or absence of a second kidney, it follows that it is of the highest importance to be able to estimate the amount of work which each kidney is doing. This can be done by catheterising the ureters, or by the separation of the urine in the bladder by a segregator. Of these two methods the former is the more reliable, though it is more difficult to carry out; the separation is complete, and a lesion of the bladder wall cannot be mistaken for one affecting the kidney, as may happen if a segregator be used, while the latter is more painful and annoying to the patient. The objections usually urged against catheterisation of the ureters are (1) that it is unsafe, (2) that it is difficult to perform. Dr. Lower states that in upwards of 50 cases which he has observed there have been no symptoms of any infection, and that in not more than 15 per cent. was he unable to catheterise the ureters.

Having obtained the urine by this method from each kidney it is necessary to make an estimate of the secretory powers of the two kidneys, and this can best be done by cryoscopy. The normal freezing point of the urine is $-1°$ to $-2·3°$. If the freezing point remains within normal limits in a case of unilateral nephritis, the affected kidney can be removed without apprehension. In proportion, however, as the freezing point of the urine approaches zero the dangers of operation will increase.

[1] Medical News, Dec. 19th, 1903.

